# New Insights into the Culture Method and Antibacterial Potential of *Gracilaria gracilis*

**DOI:** 10.3390/md16120492

**Published:** 2018-12-07

**Authors:** Gioele Capillo, Serena Savoca, Rosaria Costa, Marilena Sanfilippo, Carmen Rizzo, Angelina Lo Giudice, Ambrogina Albergamo, Rossana Rando, Giovanni Bartolomeo, Nunziacarla Spanò, Caterina Faggio

**Affiliations:** 1 Dipartimento di Scienze Chimiche, Biologiche, Farmaceutiche ed Ambientali (ChiBioFarAm), University of Messina, Viale Ferdinando Stagno d’Alcontres 31, 98168 Messina, Italy; gcapillo@unime.it (G.C.); ssavoca@unime.it (S.S.); msanfilippo@unime.it (M.S.); carmen.rizzo@unime.it (C.R.); alogiudice@unime.it (A.L.G.); 2 Dipartimento di Scienze Biomediche, Odontoiatriche, e delle Immagini Morfologiche e Funzionali (Biomorf), University of Messina, Via Consolare Valeria 1, 98100 Messina, Italy; costar@unime.it (R.C.); rrando@unime.it (R.R.); gbartolomeo@unime.it (G.B.); spano@unime.it (N.S.); 3 Istituto per le Risorse Biologiche e le Biotecnologie Marine (IRBIM-CNR), Consiglio Nazionale delle Ricerche, Spianata S. Raineri 86, 98122 Messina, Italy; 4 Science4Life s.r.l., A Spin-off of the University of Messina, V.le Annunziata, 98100 Messina, Italy

**Keywords:** *Gracilaria gracilis*, aquaculture, algal extracts, total soluble carbohydrate content, total phenolic content, single polyphenols, fatty acid composition, antibacterial activity

## Abstract

Enormous marine biodiversity offers an endless reservoir of chemicals for many applications. In this scenario, the extraction of seaweeds represents an interesting source of compounds displaying antimicrobial activity. In particular, among the different red algae, *Gracilaria gracilis* plays an important role due to the presence of important bioactives in its composition. In spite of these features, an efficient culture system is still absent. In the present study, a novel algal culture method was developed and compared to another more common cultural practice, widely reported in literature. A higher efficiency of the new method, both for daily growth rate and biomass, was assessed. Furthermore, the growth inhibitory activity of five extracts, obtained using ethanol, methanol, acetone, chloroform or diethyl ether as a solvent, from the cultured *G. gracilis* was tested against Gram-positive and Gram-negative pathogens. Algal extracts exhibited a considerable inhibitory activity against *B. subtilis* strains, while a slight inhibition was observed against *V. fischeri*. The different extracts showed significant differences in bacterial growth inhibition, with the highest activity that was recorded for the ethanol extract, followed by that of methanol. Based on the chemical characterization, these findings could be related to the antimicrobial activity played by the combination of total carbohydrates and polyphenols, which were determined at high levels in ethanol and methanol extracts, as well as by the highest number and levels of single polyphenols. Conversely, the lower growth inhibitory activities found in chloroform and diethyl ether extracts could be related to the isolation of minor lipid classes (e.g., neutral and medium polar lipids) composed by fatty acids, such as stearic, oleic and arachidonic acids, typically characterized by antimicrobial activity. In consideration of the results obtained, the present study has a double implication, involving both the field of cultural practices and the exploitation of natural sources for the isolation of antimicrobial agents useful both in pharmaceutical and food applications.

## 1. Introduction

The current application of chemical compounds isolated from diverse classes of algae and plants is actually enormous [1,2,3,4,5,6,7,8]. Recent trends in drug research from natural sources suggest that algae, in particular, are promising for the discovery of novel biochemically active substances [9,10]. Among algae, the genus *Gracilaria* (Gracilariaceae, Rhodophyta) has achieved economic importance for being a precious source of agar [11,12,13,14,15], and world-wide distributed species, such as *G. gracilis* (Stackhouse) Steentoft, Irvine and Farnham and *G. chilensis* Bird, Mc Lachlan and Oliveira [16], are characterized by the most interesting agar yield and quality [11].

Beside the important and predominant composition in agar, *Gracilaria* spp. constituents include carbohydrates, proteins, lipids, minerals and several other bioactive compounds [17,18]. These latter include, but are not limited to, polyphenols, alkaloids, polyketides, cyclic peptides, phlorotannins, sterols, diterpenoids, quinones and glycerols [19]. Such a wide array of bioactive compounds make red algae, including *G. Gracilis*, a multi-product source with various applications of great interest [17,20,21], justifying the overexploitation of the algal natural beds, and the worldwide depletion of natural populations as well. Against this backdrop, the development of high efficiency culture methods is now mandatory.

The aims of the present study were (i) to establish the efficiency of a new culture technique for *G. gracilis*, and (ii) to explore this cultivable rodophyta as a natural source of antibacterial compounds. For these reasons, two different culture methods were evaluated: one already reported in literature for another *Gracilaria* species [22] and an innovative one for *G. gracilis*. Additionally, different extracts of the cultured *G. gracilis* were prepared to test the growth inhibitory activity against Gram-positive and Gram-negative pathogens. *G. gracilis* extracts were also characterized in terms of total soluble carbohydrate content (TSCC), total phenolic content (TPC), single polyphenols and fatty acid (FA) composition, in an attempt to reveal the compounds potentially responsible for the antibacterial activity of such seaweed.

Overall, the present study may provide new insights on the aquaculture production of the red alga *G. gracilis*, and on its chemical composition as well. It may also provide a basis for practical applications not only in the aquaculture context, but also in the pharmacological and food fields.

## 2. Results

The results of the present study will be presented in different sections, according to:

- Algal culture methods;
- Antibacterial activities of algal extracts;
- Chemical characterization of algal extracts.

### 2.1. Culture Methods

**Figure 1:**
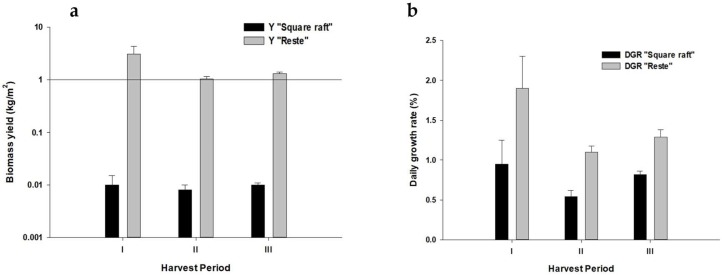
Comparison between square raft and reste culture method (**a**) Y: biomass yield (kg/m^2^); (**b**) DGR: daily growth rate (%). Culture periods: I, 15 January–15 April; II, 15 May–15 August; 15 September–15 December.

The two tested culture methods showed significant differences (*p* < 0.05) both for Biomass Yield (Y) and daily growth rate (DGR). Figure 1a,b report the overall comparison between the two methods by considering all data from the three harvest periods and the three sampling stations.

#### 2.1.1. Square Raft Method

**Figure 2:**
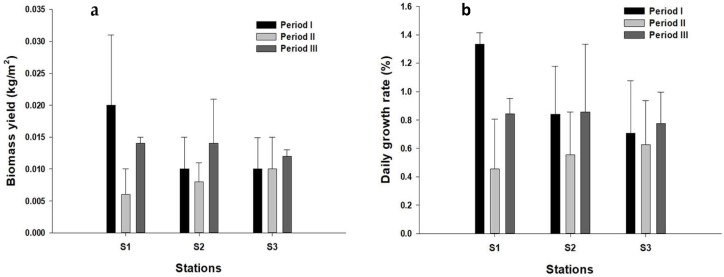
Growth of *G. gracilis* in its natural environment using square raft culture method (**a**) Y: biomass yield (kg/m^2^); (**b**) DGR: daily growth rate (%).

Both the highest and lowest biomass yield value (i.e., 0.02 ± 0.011 and 0.006 ± 0.004 kg/m^2^, respectively) were observed at station S1 in the first (January to April) and second (May to August) harvest period, respectively (Figure 2a). The results of analysis of variance (ANOVA) showed significant variations in biomass values among seasonal periods (df = 2; F = 4.211; *p* = 0.027). A post hoc Tukey’s test showed that biomass values were significantly different between the first and second period (*p* = 0.022). ANOVA did not show any significant difference between the different stations (*p* > 0.05).

The maximum DGR (1.33 ± 0.08% day^−1^) was recorded during the first culture period (January to March) at station S1, while the minimum value (0.45 ± 0.35% day^−1^) was registered during the second culture period (May to August) at the same station (Figure 2b). The results of ANOVA showed significant variations in DGR among the harvest period (df = 2; F = 3.914; *p* = 0.034). A post hoc Tukey’s test showed that DGR value was significantly different between the first and the second culture period (*p* = 0.029). There was no significant variation of DGR among the three stations (ANOVA, *p* > 0.05).

Pearson correlation analysis revealed that biomass yield had significant positive correlation with DGR (r = 0.845; *p* = 0.001), N-NO_3_ (r = 0.517; *p* = 0.006) and a significant negative correlation with temperature (r = −0.439; *p* = 0.02). DGR had a positive correlation with N-NO_3_ (r = 0.34; *p* = 0.045) and a significant negative correlation with temperature (r = −0.478; *p* = 0.012).

#### 2.1.2. ‘Reste’ Method

**Figure 3:**
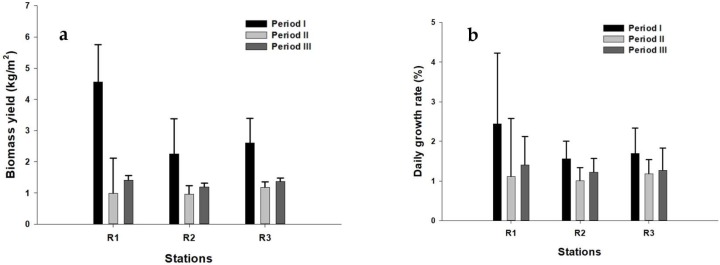
Growth of *G. gracilis* in natural environment using ‘reste’ culture method (**a**) Y: biomass yield (kg/m^2^); (**b**) DGR: daily growth rate (%).

The highest biomass yield value (4.56 ± 1.19 kg m^−2^) was observed in the first harvest period (January to March) at station S1, while the lowest biomass value (0.96 ± 0.27 kg m^−2^) was detected in the second harvest period (May to August) at station S2 (Figure 3a). The results of ANOVA showed highly significant differences in the biomass values among the seasons (df = 2; F = 14.771; *p* = 0.001). The post hoc Tukey’s test showed that biomass values were significantly different between the first and second harvest period (*p* = 0.001) and between the first and third harvest period (*p* = 0.001). No significant differences were detected among the stations (ANOVA, *p* > 0.05).

The maximum DGR (2.44 ± 1.79% day^−1^) was recorded during the first harvest period (January to March) at station S1, while the minimum value (1.01 ± 0.33% day^−1^) was registered during the second harvest period (May to August) at station S2 (Figure 3b). There was no significant variation of DGR among harvest periods and stations (ANOVA, *p* > 0.05). Pearson correlation analysis revealed that the biomass yield had significant positive correlation with DGR (r = 0.607; *p* = 0.001), N-NO_3_ (r = 0.544; *p* = 0.003) and a significant negative correlation with temperature (r = −0.572; *p* = 0.002). DGR had a significant negative correlation with temperature (r = −0.425; *p* = 0.027).

#### 2.1.3. Environmental Parameters

Results of environmental parameters of lagoon waters are reported in the Table 1, Table 2 and Table 3 regarding ST1, ST2 and ST3, respectively, during the three experimental periods.

### 2.2. Antibacterial Activity

The antibacterial activity of *G. gracilis* extracts is reported in Table 4. All extracts showed an inhibitory activity against *B. subtilis*, a Gram-positive human pathogen. The best result was obtained using 200 µg of ethanolic extract (diameter of the inhibition halo: 19 ± 1 mm). Instead, the weaker inhibitory activity was shown by the methanolic extract (50 µg) with an inhibition halo of 7.6 ± 1 mm. A slight activity was also observed against *V. fischeri*. None of the algal extracts showed antibacterial effects against the other pathogens tested as a target.

The negative control did not produce any antimicrobial activity. Chloramphenicol (30 µg), used as a positive control, produced an inhibition zone that ranged between 7.6 and 19 mm against *B. subtilis*. The minimum inhibitory concentration (MIC) values of the various extracts ranged between 40 and 10 µg/disc. Each extract, tested at the same amount as the positive control, showed a lower inhibition capacity compared to this.

### 2.3. Chemical Characterization

#### 2.3.1. Total Soluble Carbohydrate (TSCC) and Total Phenolic Content (TPC)

In Table 5, a comparative evaluation of extraction yield, the TSCC and TPC of the different extracts from *G. gracilis* are reported. Results are expressed on a dry weight (dw) basis.

Overall, the ethanol extract showed the highest yield (9.80%) and TSCC (553.24 g GE/Kg, *p* < 0.05), and a high TPC (2059.79 mg GAE/100 g, *p* < 0.05) as well. Next up, the methanol extract was characterized by lower yield (1.37%) and TSCC (159.14 gGE/Kg, *p* < 0.05), but, at the same time, by the highest TPC (2741.93 mg GAE/100 g, *p* < 0.05). Apolar solvents, such as chloroform and diethyl ether, showed a similar behavior as they provided the lowest extraction yields (0.61% and 0.39%), as well as the lowest TSCC (0.39 and 0.35 g GE/Kg, *p* > 0.05) and TPC (35.90 mg GAE/100 g and 28.83 mg GAE/100 g, *p* > 0.05).

Although the acetone extract showed a yield (0.51%) similar to the apolar solvents, intermediate TSCC (42.83 g GE/Kg, *p* < 0.05) and TPC (810.32 mg GAE/100 g, *p* < 0.05) with respect to the other extracts were obtained.

#### 2.3.2. Single Polyphenols

The amounts (mg/100 g) of single polyphenols detected in the dried algal material by reversed phase ultra-high performance liquid chromatography coupled with diode-array detection (RP-UHPLC-DAD) are shown in Table 6. Overall, the ethanol and the methanol extracts were characterized by the same compounds, in terms of phenolic acids, flavonoids and catechins. Nevertheless, almost every polyphenol resulted in being at higher concentration in the methanol extract than the ethanol extract (*p* < 0.005). The acetone extract showed a similar qualitative profile, but a lower number of phenolic acids was detected. Additionally, almost every compound was found at lower concentration (*p* < 0.05) with respect to the other *G. gracilis* extracts.

In greater detail, among the phenolic acids, the protocatechuic and 4-hydroxybenzoic acids were characterized by the highest levels (103.76–561.00 mg/100 g and 59.36–167.00 mg/100 g, respectively) in each type of extract; whereas rutin and hesperidin were the most abundant flavonoids (140.70–334.23 mg/100 g and 203.43–741.30 mg/100 g, respectively). Interestingly, two catechins, namely epicatechin and epicatechingallate, were also revealed. Nevertheless, by contrast with the other polyphenols, such compounds were found at the highest levels (*p* < 0.05) in the acetone extract (23.31 mg/100 g and 35.16 mg/100 g, respectively).

#### 2.3.3. Fatty Acid Composition

Table 7 reports the percentage of fatty acid methyl esters (FAMEs) revealed in both chloroform and diethyl ether extracts obtained from the dried *G. gracilis*. Gas chromatography–flame ionisation detection (GC-FID) analyses allowed to determine 17 and 16 FAMEs in chloroform and diethyl extracts respectively, ranging from lauric acid methyl ester (C12:0) to tetracosanoicacid methyl ester (C24:0). Overall, both extracts were characterized by a similar qualitative profile, as the only differences were the absence of docosanoic acid methyl ester (C22:0) and eicosadienoicacidmethyl ester (C20:2n-6) in the diethyl ether extract, and the absence of docosahexaenoic acid methyl ester (C22:6n-3) in the chloroform extract as well. From a quantitative point of view, non-significantly different contents (*p* > 0.05) were found between chloroform and diethyl ether extracts for almost each FAME. The only exceptions were represented by palmitic acid methyl ester (C16:0) (respectively 31.62% and 41.71%, *p* < 0.05), oleic acid methyl ester (C18:1n-9) (respectively 10.33% and 9.21%, *p* < 0.05) and arachidonic acid methyl ester (C20:4n-6) (respectively 38.33% and 29.40%, *p* < 0.05). Also, these three FAMEs were the most abundant compounds respectively among saturated fatty acids (SFAs), monounsaturated fatty acids (MUFAs), and polyunsaturated fatty acids (PUFAs) in both extracts.

However, diethyl ether extract was generally characterized by a higher SFA content than chloroform extract (52.81% vs. 42.36%), whereas the chloroform extract showed higher MUFA and PUFA contents when compared to the diethyl ether counterpart.

## 3. Discussion

Gracilariales are efficient bio-filters for aquaculture waste water treatment, removing nitrogen and phosphorous salts [23,24,25], and also critical rare elements [26]. For these reasons, they represent good candidates for integrated multitrophic aquaculture (IMTA). Among them, *G. gracilis* possesses a further potential in the aquaculture industry due to its high-quality agar content respect to other agarophytes [27,28,29]. Moreover, *G. gracilis* is a well-known source of bioactive compounds, such as lipids, fatty acids, sterols, phenols and others [17,30,31]. All these features, combined with the growing depletion of natural beds of *Gracilaria* [14] and the scarce available literature on culture methods [32], create the basis for the present study, which was aimed at improving the current knowledge and the know-how of *G. gracilis* culture and exploring the antibacterial potential of its extracts.

Cultivation of seaweed is an old practice that has encompassed numerous evolutions and modifications [33]; those for the genus *Gracilaria* have been recently reviewed by Capillo et al. [20]. A diffused and studied red algae culture method is the raft culture [22,25]. In the present study, the raft culture method was used for the experimental cultivation of *G. gracilis* in the brackish water of the natural reserve of “Capo Peloro” (Italy). Raft culture methods was compared with an innovative method, designed by the authors, consisting of nylon nets, the “reste” (the same used for mussel rearing), in which thalli of algae were inserted. The “reste” culture method showed the highest efficiency both in term of biomass and DGR. This result was strictly related to the culture method itself as other factors possibly influencing algal growth (e.g., depth of implants [34], different stations and seasons [29]) did not seem to affect the algal growth. It is conceivable that the structure of culture implants significantly affected the growth rate and the biomass yield. The “reste” method had a three-dimensional structure thanks to its cylinder geometry; this allows the thalli to grow in all directions, giving a major volume to the possible algal growth.

Using both culture methods *G. gracilis* growth showed clear seasonal trends with higher values in the winter season. Factors affecting *Gracilaria* growth are various and include irradiation, salinity, temperature, dissolved oxygen, and dissolved nutrient availability [35]. Our findings are in accordance with those obtained by Polifrone et al. [13] who reported the higher concentration of *G. gracilis* natural bed during the winter season, but they are in contrast with observations made in other world regions, such as Turkey and Argentina [14,32]. Similar results were reported for the congeneric *G. edulis* along the south-eastern coast of India and Gulf of Mannar [22,36,37]. The growth of *G. gracilis* was lower in the second culture period, from May to August, confirming previous results for the same area [38]. Statistical elaborations confirmed a significant negative correlation between growth (for both culture methods) and temperature. Despite the confirmed vulnerability of the alga to the high temperature [39], the cultured alga showed slight growth also in the summer period. This result furnishes an important input to try producing such seaweed also in summer, in contrast with previous statement of *G. gracilis* absence in this period of the year [38]. No significant differences among the three stations were noted. This result confirms the stable status of the brackish lagoon water in which experimental cultures were allocated [40].

The covering of seaweeds by other algal species represents one of the problems in algal culture. In our case, the negligible presence of other seaweeds derives from the suspended position of the implants, the poor but efficient surf action, and the fact that *G. gracilis* in brackish water is able to form a temporal monodominant community releasing in the water substances able to inhibit the growth of other algal species [39].

A sustainable and continuous cultivation of *G. gracilis* may support the exploitation of its great biotechnological potential. Such red alga has been already explored for a wide variety of chemical compounds, and for various bioactivities as well, making it a valuable resource [41]. Nonetheless, a literature review has highlighted that few studies have explored the antibacterial activity of this seaweed, and the potential compounds responsible of such activity as well [42,43]. Therefore, considering the importance of algal bioactive compounds, as an excellent alternative to the current antibiotics on the market, as well as the easy availability of the algal species in question, it was considered necessary to investigate its antimicrobial potential more in depth. In this study, the antibacterial potential of *G. gracilis* extracts was evaluated against some marine and terrestrial bacterial pathogens. In an attempt to evaluate the antibacterial capacities of the different compounds that constitute the algae, different organic solvents have been used for extraction. Our results showed that all the extracts obtained had antibiotic activity against *B. subtilis*. This result, according to what is reported by other authors, shows that Gram positive bacteria are more susceptible to red algae extracts, probably due to reasons related to the cell wall composition of these bacteria or hydrophobicity of the extracted compounds [44]. Ethanol extract produced the greater inhibition halo, while the extracts obtained through the use of acetone, diethyl ether and chloroform have shown a more similar and low inhibitory efficacy. The extracts of *Gracilaria* spp., obtained employing acetone, hexane and ethanol, have been shown to have a good inhibitory power against Gram positive and gram negative pathogens [45,46]. In particular, ethanolic and water extracts obtained from *G. gracilis* has already exhibited inhibitory activity against *Vibrio harveyi*, *Vibrio cholerae* and *Aeromonas hydrophyla*, demonstrated by the presence of an inhibitory halo [43]. In our case, the inhibitory activity was exhibited against *B. subtilis*, especially with ethanol extract. Notwithstanding this, several factors, as well as water parameters and algae physiological conditions, could determine a variability in the bioactive compounds production by seaweeds, even considering the same algal genus [41].

To comprehend the biological activity of *G. gracilis* in depth, a characterization of the principal compound classes potentially responsible for the antibacterial properties of such seaweed was carried out. Organic solvents with different polarity indices were exploited as extracting media so that the potentially bioactive compounds, marked by peculiar chemical properties, and polarities as well, could be isolated as exhaustively as possible. Overall, as expected, the results obtained highlighted that the type of solvent was responsible for peculiar characteristics of each extract, in terms of extraction yield, TSCC, TPC, levels of single polyphenols and FA composition, the extraction method being equal. Once again, this points out that the isolation of (bioactive) compounds is significantly affected by the nature of solvent used and their polarity as well [43,44].

As shown in Table 2, the yield of the extracts considered decreased in relation to the decreasing polarity of solvents. A similar behavior was observed also for TSCC and TPC data. In the present study, the TSCC of each extract was investigated since red algae represents one of the most important sources of non-animal sulfated polysaccharides, notoriously characterized by antitumoral, antioxidant, antiviral activities and, not least, antimicrobial properties [47,48]. As the carbohydrate fraction is typically highly soluble in polar solvent such as ethanol [49,50,51], the highest TSCC was found in the ethanol extract, whereas slightly less polar solvents, such as methanol and acetone, showed lower contents (Table 2). On the other hand, the TSCC revealed in extracts obtained by means of apolar solvents, namely chloroform and diethyl ether, was inferior to 100 g GE/Kg. Few previous studies investigated the content of total carbohydrates in *Gracilaria* spp., reporting not comparable results. In a work conducted by Manivannan and colleagues [52], *G. folifera* from the southeast coast of India reported a TSCC of 223.2 g/Kg (dw). In another study, *G. verrucosa* collected in the Sea of Marmora had a TSCC equal to 43.07 g/Kg (dw) [53]. Only a recent investigation conducted by Francavilla and coworkers [17] on *G. gracilis* from the Mediterranean Lesina lagoon (Italy) highlighted slightly lower TSCCs, which varied depending on the sampling season (from 347 g/Kg to 248 g/Kg, dw).

Polyphenols are the most widely distributed secondary metabolites, ubiquitously present in the plant kingdom, although the type and content of every compound may vary according to the phylum, and even the species, taken into consideration [54,55,56]. Red algae are known to produce a wide array of polyphenols which, similar to carbohydrates, have been studied in depth for their antioxidant [57], antitumoral [58], and, above all, antibacterial activity [45,59]. Hence, the TPC was reasonably investigated in each extract subjected to antimicrobial assays, and the polyphenol profile of the extracts with the highest phenolic content was explored.

Due to the huge chemical complexity of the polyphenol class, the extensive scientific literature has already demonstrated that an exhaustive extraction method can hardly be developed. Also, the extraction procedure becomes more challenging when such compounds are naturally complexed with other molecules such as proteins, polysaccharides, and lipids [60]. However, it is well accepted that, owing to their phenolic (hydrophilic) nature, polyphenols can be readily isolated by polar solvents, including alcohols, such as methanol, ethanol, and ketones, such as acetone [61].

Against this backdrop, it is evident that the TPC showed significant differences (*p* < 0.05) in almost each extract according to the polarity of the solvent used. Indeed, the highest TPC was obtained with ethanol and methanol, followed by acetone (Table 1). In the ethanol and methanol extracts it is supposed that we can find mainly, but not only, lower molecular weight polyphenols [61]; whereas; higher molecular weight polyphenols may be mainly, but not only, isolated in the acetone extract [62]. The lowest and non-statistically significant different (*p* < 0.05) TPCs found in the chloroform and diethyl ether extracts could be due to a lower solubility of polyphenols in such apolar solvents. Overall, the TPC data obtained in the present study are hardly comparable with previous data reported on *Gracilaria* spp. For example, the dried *G. changii* collected in the mangrove area of Santubong (Malaysia) had TPCs equal to 1083 mg GAE/100 g, 858 mg GAE/100 g, and 820 mg GAE/100 g, respectively, in ethanol, methanol and acetone extracts [63]. *G. vermiculophylla* from the coastal areas of Denmark showed TPCs of 51.4 mg GAE/100 g and 95.2 mg GAE/100 g on a dw basis, respectively in water and ethanol extracts [64]; whereas the methanol extract of *G. edulis* collected in Tamil Nadu (India) reported a total amount of phenolic compounds equal to 32,700 mg GAE/100 g. *G. gracilis* coming from the West coast of Ireland showed TPC data lower than those reported in the present study, as aqueous methanol and aqueous ethanol extracts were respectively characterized by TPCs equal to 536 mg GAE/100 g and 476 mg GAE/100 g [65]. However, concerning the directly proportional relation between TPC and solvent polarity, contrasting results were revealed by a recent work [17] focused on the chemical characterization of the dried *G. gracilis* from the Mediterranean Lesina lagoon (Italy). In this study, TPCs of extracts obtained by ethyl acetate extract, hexane and methanol were around 4500–6500 mg GAE/100 g, 1156–3180 mg GAE/100 g, and 230 mg GAE/100 g, respectively.

Since only three of the five extracts from *G. gracilis* reported considerably high TPCs (Table 2), ethanol, methanol and acetone extracts were screened for single polyphenols.

To the best knowledge of the authors, this is the first report on single polyphenols from *G. gracilis*. Although different works were previously carried out on the polyphenol fingerprints of different species of red algae [66,67], the few studies focused on *Gracilaria* spp. [64,68] make comparison with the present results, once again, difficult. Farvin and Jacobsen [64] highlighted in the Danish *G. vermiculophylla* the presence of only three phenolic acids (gallic, protocatechuic and gentisic acids), confirming protocatechuic acid as the most abundant. Nonetheless, such a polyphenol was found at much higher levels (2920 mg/100 g, dw) with respect to the amount detected in this study. Yoshie-Stark et al. [68] investigated the flavonoid profile of different Japanese seaweeds, and species such as *G. texorii* and *G. asiatica* were characterized by much higher and not comparable amounts of quercitin (3000 mg/100 g and 2050 mg/100 g, on a dw basis) and hesperidin (11,900 mg/100 g and 11,200 mg/100 g, on a dw basis). However, no quercitin and myricetin were revealed.

In the same species, catechins such as epigallocatechin (890 mg/100 g and 1100 mg/100 g, dw) and epigallocatechingallate (240 mg/100 g and 180 mg/100 g, dw) were found [69]. Also in this case, much higher, and not comparable, levels of such phytochemicals were highlighted with respect to data reported in the present study.

In line with TPC results, the ethanol and methanol extracts showed a higher number and content of single polyphenols than the acetone extract. This could explain the highest antibacterial activities revealed by the same extracts against the food-borne pathogen *B. subtilis*. Additionally, coherently with TSCC results, it could be hypothesized that a synergistic action of polyphenols and carbohydrates occurred mainly in the ethanol extract, being responsible for the intensified antibacterial activity of such an extract. Similar results were also observed for different *Gracilaria* species, namely *G. corticata*, *G. folifera*, *G. edulis*, *G. debilis* and *G. crassa*. Overall, crude ethanol and methanol extracts of such algae reported higher growth inhibitory activities against human pathogens, including *B. subtilis*, then extracts obtained by less polar solvents [70,71]. Nonetheless, any chemical characterization of such bioactive extracts was carried out in these previous works.

In the present study, the FA composition of *G. gracilis* was explored as, similar to carbohydrates and polyphenols, certain fatty acids from red algae result to be characterized by antimicrobial activity [41,72]. However, only the chloroform and diethyl ether extracts were taken into consideration, as apolar compounds, such lipids, show high affinity with apolar solvents.

Although cellular total lipids are typically isolated by a mixture of apolar–polar solvents, such as chloroform and methanol, with various ratios (2:1 or 1:1) [73,74], previous studies reported the use of chloroform and diethyl ether for the isolation of neutral lipids (e.g., triglycerides, diglycerides, monoglycerides, cholesterol, and cholesterol esters) and free fatty acids, respectively [75,76,77]. Consequently, in the present study, chloroform and diethyl ether allowed to isolate only such minor lipid fractions, being responsible for the lower extraction yields when compared not only with the other extracts investigated in this study (Table 1), but also with the total lipid yields of dried material from *G. gracilis* previously investigated [17,78].

The FA composition of the extracts under investigation was quite similar to those from previous studies focused on dried material from *G. gracilis*. In fact, palmitic acid was the most abundant saturated fatty acid also in the red alga from the Mediterranean Lesina lagoon (25–38%) [17] and the Irish coast (39%) [78]; whereas oleic acid (5.76–10.78% and 8.4%) and arachidonic acid (16–47.78% and 30.8%) confirmed to be the predominant fatty acids respectively in the MUFA and PUFA fractions of such algae. Also the sum of SFAs, MUFAs and PUFAs was within the range of data reported in these previous works [17,78].

The weak growth inhibitory activity against *B. subtilis* showed by the chloroform and diethyl ether extracts of *G. gracilis* was also observed in low polar and apolar extracts of *Gracilaria* spp. Indeed, diethyl ether extracts of *G. crassa*, *G. folifera*, *G. debilis* and *G corticata* showed lower antibacterial activities against different pathogens, including *Bacillus* spp., when compared with extracts obtained from more polar solvents [79].

However, the antibacterial activities reported by such extracts could be justified by the presence of certain fatty acids characterized by antimicrobial activity. Indeed, Manilal and colleagues [72] reported that fatty acids, such as stearic (C18:0) and oleic (C18:1n-9) acids, were found in the bioactive fraction the red algae *Asparagopsis taxiformis*. Also, long chain fatty acids, including arachidonic acid (C20:4n-6) found at high levels, seem to stimulate oxygen uptake by Gram-positive bacteria at bactericidal concentrations [80].

## 4. Materials and Methods

### 4.1. Study Area, Collection and Implant Sites

The study was carried out in the Ganzirri lagoon (Eastern Sicily, Italy; 38°24′ N; 15°62′ E), characterised by brackish waters of marine origin [81].

The lagoon has a surface of 34 ha and entire volume 9.8 × 105 m^3^ with a 6.5 m maximum depth. It has the appearance of a long (1670 m) and narrow (on average ∼200 m) stream tube parallel to the coast, and it is characterized by a slightly low salinity (on annual average 31, ranging from 21–37) [82,83], and water temperatures with annual average about 23.1 °C as well. The salinity values have great oscillations: in winter the lagoon reaches hypoaline values (21 PSU) due to high rainfall, while in summer up to 39 PSU.

The Ganzirri is characterised by two sub-basins: north and south. The southern basin (3 m average depth) has been extensively exploited for over a century for molluscs’ culture (mussels and clams), has muddy sediments, and primary production is sustained essentially by phytoplankton [84]. The north basin accounts for one quarter of the total surface area, is shallower (maximum depth 1 m) than the southern one, and has sandy bottoms and mats of the green alga *Chaetomorpha* spp. Primary production in the north basin is due both to phytoplankton and green and red macroalgae. The two Ganzirri basins, being partially separated from each other by a sand tombolo, are characterised by different hydrodynamic regimes [85]. The lagoon is connected with both the Ionian Sea and Faro lake by the Torri-Catuso and Margi channels, respectively. The Margi channel is about 1 km long and 10–12 m large.

### 4.2. Algal Collection

Samples of the rhodophyta *Gracilaria gracilis* (Stackhouse) Steentoft, Irvine and Farnham were collected between the northern part of the lagoon and the Margi channel. Samples of algae were cleaned grossly in the water of lagoon and transferred to the aquaculture laboratory of the University of Messina (Italy) in a special polyethylene transport tank (Narvalo model 120 × 100 × 90 cm, INNOVAQUA, Cadelbosco Sopra, Reggio Emilia, Italy). Algae were carefully observed, cleaned, separated from other algal species and epiphytes to allow a mono-specific culture.

### 4.3. Culture Methods

Two different culture methods were tested: square raft and “reste” method. The first method consists of square raft (90 × 90 cm) of pine wood strips of 5 × 2 cm. Each raft held 8 parallel lines of polypropylene rope (3 mm) where vegetative fragments of thalli were inserted (Figure 1). The reste method, developed by the authors, consisted of a nylon net where thalli were inserted using a 5-cm diameter PVC tube to open the net. A3-cm diameter PVC tube was used as a piston to push algae inside the net. The total length of each resta was 4 m. Both square frames and reste were anchored securely by stones and signalled on the surface by marker buoys.

### 4.4. Culture Experimental Design

The study was carried out from January to December 2017. Square raft and reste, previously prepared, were allocated in three different sites of the Ganzirri lagoon, named S1 (38°25′90″ N; 15°61′47″ E), S2 (38°26′15″ N; 15°62′10″ E) and S3 (38°26′11″ N; 15°61′52″ E). Three culturing periods were evaluated in order to compare the seasonal variation in growth: I, 15 January–15 April; II, 15 May–15 August; 15 September–15 December.

### 4.5. Environmental Factors

Water parameters were determined monthly throughout the culture periods. Temperature and salinity were measured with a multiparametric probe (YSI30, Yellow Spring Incorporated, OH, USA), pH with a pHmeter (pH110, XS Instruments, Singapore). Oxygen was measured using a multi-parametric probe. In order to calibrate the oxygen sensor, the Winkler method was used for the determination of DO [86]. Triplicate seawater samples were collected from the implantation sites monthly and analysed for nutrients. N-NO_2_, N-NO_3_, P-PO_4_ and N-NH_4_ were determined using an ultraviolet (UV) spectrophotometer (Shimadzu UV-1800, Shimadzu Corporation, Kyoto, Japan) according to standard methods [87].

### 4.6. Growth Rate

Growth was measured by gravimetric methods. Frames and the reste were collected monthly from the field and transferred to in-shore plants to evaluate the condition of the culture. Square frames method: each square’s rope was weighted by electronic balance FX-3000 (Max 3100g precision d = 0.01 g, A&D, 1756 Automation Parkway, San Jose, CA, USA). Reste method: each resta was weighted by a commercial balance. Before weighing the algal cultures were washed thoroughly in lagoon water. All foreign bodies and attached flora and fauna were removed manually. Excess water was drained by hanging plants horizontally for 15–20 min.

The daily growth rate (DGR) (%/day) was calculated using the formula of Dawes et al. [88] as follows:

(1) $$\mathrm{DGR}\left(\frac{\%}{\mathrm{day}}\right)=\ln\left(W_{f}/W_{i}\right)/t\times 100$$

where *W_f_* is the final fresh weight after *t* days of culture period and *W*_0_ is the initial fresh weight.

Biomass (*Y*) expressed as mean kg fresh wt m^−2^ was determined using the modified formula of Doty [89] that included the initial weight of the propagules as follows:

(2) $$Y=\frac{\left(W_{f}-W_{i}\right)}{m^{2}}$$

where *W_f_* is the final fresh weight and *W*_0_ is the initial fresh weight. This was used for *Y* determination of square frame method. Instead, in order to assess the biomass yield of the “reste” method, at (2) it is important to apply a correction factor to allow comparison of two different methods. In fact, the reste method involves the use of cylindrical structures and, for this reason, to bring the volume of a cylinder to a surface, the simple formula below was used:

(3) $$S_{c}=2\pi rh$$

where *S_c_* is the surface of the “open” cylinder, *r* is the radius, and *h* is height, which inserted in place of m^2^ in (2) gives:

(4) $$Y=\frac{\left(W_{f}-W_{i}\right)}{2\pi rh}$$

### 4.7. Chemicals and Reagents

Organic solvents, such as methanol (high-performance liquid chromatography (HPLC) grade, Carlo Erba, Val de Reuil, France), ethanol (absolute, VWR International, Fontenay-sous-Bois, France), acetone (reagent grade, Sigma-Aldrich, St. Louis, MO, USA), chloroform (VWR International, Fontenay-sous-Bois, France) and diethyl ether (reagent grade, VWR International, Fontenay-sous-Bois, France), were employed for the different extraction procedures. For the assessment of total soluble carbohydrates and polyphenol contents, concentrated sulfuric acid and phenol (reagent grade) were from J.T. Baker (Phillipsburg, NJ, USA), whereas Folin–Ciocalteu reagent, D-glucose, and gallic acid were purchased from Sigma-Aldrich (Milan, Italy). For single polyphenol analysis, commercial standards were supplied by Sigma-Aldrich (Milan, Italy). For the determination of fatty acid composition, *n*-hexane was purchased from PanReacAppliChem (Barcelona, Spain), whereas fatty acid methyl ester (FAMEs) reference standards (C4-C24) were from Supelco (Bellefonte, PA, USA).

### 4.8. Extraction Methods

To explore the effectiveness of different extraction methods on the antibacterial activity of *G. gracilaris*, four different sample preparation procedures were carried out by employing five solvents, namely methanol, ethanol, chloroform, acetone and diethyl ether. In each case, algal raw material (collected at the end of the last culture period) was shade dried at room temperature and, subsequently, ground with the help of a mortar and pestle. Powdered algal samples were stored at 4 °C until solvent extraction. For the extraction processes, ~40 g of powdered algal material was extracted with 200 mL of organic solvent by means of a Soxhlet apparatus. The obtained extract was then filtered and concentrated to dryness by a rotating evaporator (Rotavapor Büchi V700, BUCHI Labortechnik AG, Flawil, Switzerland). The extraction yield, expressed in percentage, was calculated as follows:

Yield (%) = (*W*_1_/*W*_2_) × 100

where *W*_1_ is the weight of the dried extract, and *W*_2_ is the weight of the dried algal material. The dried extracts werestored at 4 °C until analysis.

### 4.9. Evaluation of Antibacterial Activity

The antibacterial potential of the five extracts of *G. gracilis* was evaluated against both Gram-negative (i.e., *V. cholerae*, *Pseudomonas aeruginosa*, *Salmonella* sp., *Aeromonas hydrophila*, *V. fischeri*) and Gram-positive (*B. subtilis*) pathogens.

The agar disk diffusion method (Kirby Bauer test) [43] was used, according to the National Committee for clinical Laboratory Standards [90]. Briefly, target strains were prepared to obtain a working culture containing approximately 1 × 10^8^ cells/mL: marine pathogens were cultivated on nutrient broth (NB; Oxoid) amended with 3% NaCl at 25 °C for 18–24 h; terrestrial pathogens on NB at 37 °C for 18–24 h. Aliquots (100 µL) of each bacterial suspension, were inoculated using spread plate method on Tryptone Soy Agar (TSA, Oxoid) amended with 1.5% NaCl.

To perform the test, 10 mg of each extract were dissolved in 1 mL of the respective solvent and diluted to obtain three different dried extract amounts (i.e., 50, 100, 200 µg) in 20 µL to be used to soak sterile disks (6mm in diameter, Oxoid). After 48 h, allowing the complete evaporation of the solvent, the disks were applied to the inoculated plates. Disks containing 30 µg of chloramphenicol were used as a positive control, while disks soaked with 20 µL of the appropriate solvent were used as a negative control. The plates were incubated overnight at optimal growth temperatures for pathogens. The diameter of the inhibition zone was measured, and means and standard deviations were calculated. The antibacterial assay was carried out in triplicate.

The minimum inhibitory concentrations (MICs) of all extracts were determined by serial dilution to obtain different extract amounts (i.e., 40, 30, 20, 10, 5 µg/disk).

### 4.10. Determination of TSCC and TPC

The TSCC of each extract from *G. gracilis* was determined by the phenol-sulfuric acid method [91]. Briefly, each extract (~50 mg) was re-suspended in 1 mL of its extraction solvent by vortexing, and subsequently diluted 1:100. Then, 2 mL of the resulting solution was mixed with 100 µL of 80% phenol and 5 mL of concentrated sulfuric acid and allowed to stand for 10 min at room temperature. Subsequently, the sample was placed in a water bath at 30 °C for 20 min and, finally, the absorbance was measured at 490 nm by a ultraviolet–visible (UV–VIS) spectrophotometer (UV-2401PC, Shimadzu, Milan, Italy). For quantify the TSCC, solutions of D-glucose were employed to construct a six-point calibration plot in the range 50–5000 ppm. Consequently, the TSCC was calculated as g of glucose equivalent in 1 Kg of dried algal sample (g GE/Kg, dw).

The TPC was assessed by the method based on the use of the Folin-Ciocalteu reagent, proposed by Singleton et al. [92]. Briefly, about 50 mg of each extract were re-suspended in 10 mL of distilled water and centrifuged at 9000 rpm and 4 °C for 30 min. Then, 1 mL of the obtained supernatant was mixed with 5 mL of Folin–Ciocalteau reagent and 15 mL of sodium carbonate (20%) in a 100-mL volumetric flask, and added with distilled water up to a final volume of 100 mL. The resulting solution was kept in the dark for 120 min, and subsequently analyzed with the UV–VIS spectrophotometer (UV-2401 PC, Shimadzu, Milan, Italy). The wavelength of absorbance was set at 760 nm. A six-point calibration curve ranging from 50 to 5000 ppm was constructed using appropriate solutions of gallic acid as external standard. As a result, the TPC was calculated as mg of gallicacid equivalent in 100 g of dried algal sample (mgGAE/100 g).

In both TSCC and TPC analyses, each extract from *G. gracilis* was analyzed in triplicate.

### 4.11. Polyphenol Characterization by Ultra-High Performance Liquid Chromatography (UPLC)

RP-UPLC-DAD analyses were carried out on a Shimadzu Prominence UFLC XR system (Shimadzu, Kyoto, Japan), consisting of a CBM-20A controller, a LC-20AD-XR binary pump system, a DGU-20A_3R_ degasser, a SPD-M20A photo diode array detector, a CTO-20AC column oven and a SIL-20A XR autosampler. Data acquisition was performed by Shimadzu Lab Solution software (v. 5.53 SP2, Shimadzu, Kyoto, Japan). For the chromatographic separations, a Kinetex XB-C18 (100 mm × 2.1 mm; particle size 2.6 μm) from Phenomenex (Torrence, CA, USA) was employed. The mobile phase was composed of inorganic (water, phase A) and hydro-organic (methanol, phase B) solvents, both acidified with 0.1% formic acid. The optimized gradient program was: 0–10 min, 2–20% B; 10–18 min 20–50% B; 18–20 min 50–90% B.

The mobile phase flow rate was 0.2 mL/min, while the oven temperature and injection volume were, respectively, set at 37 °C and 2 µL. The PDA spectra were acquired in the range 190–500 nm, and the chromatograms were extracted at 280 and 350 nm (time constant: 0.64 s; sample frequency: 40 Hz). Identification of single polyphenols occurred by comparing the retention time of chromatographic peaks and the underlying PDA spectra as well from algal extracts with those from commercial standards, analyzed by the same operating conditions.

Concerning the quantification procedure, a six-point calibration curve was built up for each of the analytes investigated by means of stock standard solutions diluted in the range of 50–500 ppm (syringic, *p*-coumaric, ferulic acids, epicatechin and epicatechingallate), 100–1000 ppm (gallic, chlorogenic and caffeic acids), 500–5000 ppm (protocatechuic and 4-hydroxybenzoic acids, rutin, hesperidin, myricetin and quercetin). For each sample, three replicate determinations were carried out.

### 4.12. Analysis of the Fatty Acid (FA) Composition

The fatty acids eventually present in the algal extracts were determined by gas chromatographic quantification of their methyl esters (FAMEs), which were prepared by suspending ~50 mg of the extract in 1 mL hexane, adding the mixture with methanol/sulfuric acid (9:1), and finally heating at 100 °C for 1 h. The hydrocarbon layer was collected and injected into a Master GC-DANI system (Dani Instrument, Milan, Italy), equipped with a split/splitless injector, and a flame ionization detector (FID). A capillary column Supelco SLB-IL100 (60 m × 0.25 mm, film thickness 0.20 µm) was employed for the chromatographic separations. The oven temperature program was 120–200 °C at 1 °C/min (10 min). Injector and FID temperatures were set at 220 and 240 °C, respectively. Carrier gas was He, at a constant linear velocity of 30.0 cm/s. FID conditions were set as follows: sampling frequency: 25 Hz; gases: makeup (He), 25 mL/min; H_2_, 40 mL/min; air, 280 mL/min. Data were processed through the Clarity software (Dani Instrument, Milan, Italy).

FAMEs were identified by comparing the retention times of chromatographic peaks from samples and those from commercial standards, analyzed according the same operating conditions; whereas the quantification of individual FAMEs was calculated as percentage content in relation to the total area of the chromatogram. All determinations were run in triplicate.

### 4.13. Statistical Analyses

All data are presented as means ± standard deviations (SD), from three replicates. Statistical analyses were done using SYSTAT version 13 (Systat Software, San Jose, CA, USA).

Specifically, a one-way analysis of variance (ANOVA) was performed to determine the significance of differences in biomass yield (Y) and daily growth rate (DGR) for: different cultivation methods, stations and cultivation periods. A Tukey’s honestly significant difference (HSD) test was applied for a post hoc comparisons, when significant differences were found (*p* < 0.05). Pearson’s correlation analysis was used to correlate the relationships between Y, DGR and the influence of environmental factors.

Concerning results from chemical characterization of the extracts, data from TSCC, TPC and single polyphenols were also subjected to one-way ANOVA, followed by the Tukey’s HSD test and significance level was set at *p* < 0.05. Data from FA composition were statistically elaborated by a Student’s two-tailed *t*-test for unpaired data, and statistical significance was accepted at *p* < 0.05.

## 5. Conclusions

In order to reduce the overexploitation of *G. gracilis* natural beds, this study provides innovative and efficient culture methods for this Rhodophyta. Moreover, antibacterial assays and chemical analyses conducted in this study encourage the cultivation of *G. gracilis* for potential applications both in pharmaceutical and food areas.

Since the development of resistance to current antibiotics is continuously increasing and the production of novel bioactive compounds has decreased, thousands of marine compounds have been isolated and characterized over the last decades for developing innovative antibiotics [93]. On the basis of the results obtained, methanol and ethanol extracts from *G. gracilis* could be useful to isolate chemicals acting as antibacterial agents against *B. subtilis*, although further studies will be required to test the extracts also in vivo and to evaluate their cytotoxicity. However, *G. gracilis* could represent also an interesting source for designing antiseptic and cleansing products.

In the food industry, the bacterial contamination of foods is not yet under adequate control, and, despite the wide array of available preservation methods, the demand for non-toxic and natural preservatives inhibiting food-borne pathogens, such as *B. subtilis*, has been constantly increasing [92]. Hence, the powerful antimicrobial activities of *G. gracilis* against *B. subtilis* suggest that the seaweed extracts could be a potential natural alternative to chemical preservatives in foods. Overall, this study points out the high potential of the Mediterranean *G. gracilis* for aquaculture, pharmaceutical and, not least, food applications.

## Figures and Tables

**Table 1:** Water parameters in the three periods at ST1. The results are shown as means ± standard deviation of three replicates. T (°C), Sal (‰), O_2_ (sat%), N-NH_4_, N-NO_2_, N-NO_3_, P-PO_4_ (μM/L).

	ST1					
	I		II		III	
Parameter	Mean ± SD	Range	Mean ± SD	Range	Mean ± SD	Range
T	16.15 ± 3.13	13.27 ÷ 20.53	26.07 ± 3.52	21.23 ÷ 29.58	20.29 ± 3.86	15.63 ÷ 24.85
SAL	31.62 ± 0.46	31.2 ÷ 32.2	31.5 ± 0.94	30.9 ÷ 32.9	31.15 ± 1.09	29.9 ÷ 32.3
pH	8.66 ± 0.13	8.51 ÷ 8.84	8.49 ± 0.11	8.38 ÷ 8.63	8.39 ± 0.16	8.21 ÷ 8.59
O_2_	104.25 ± 1.03	102.8 ÷ 105.2	118.5 ± 15.94	98.1 ÷ 136.9	113.3 ± 32.16	74.8 ÷ 152.1
N-NH_4_	1.52 ± 0.31	1.12 ÷ 1.88	1.75 ± 1.16	0.66 ÷ 3.07	3.12 ± 0.92	2.04 ÷ 4.3
N-NO_2_	0.34 ± 0.22	0.14 ÷ 0.66	0.17 ± 0.12	0.04 ÷ 0.31	0.7 ± 0.72	0.2 ÷ 1.76
N-NO_3_	8.94 ± 5.69	2.79 ÷ 16.57	1.10 ± 0.75	0.15 ÷ 1.99	5.02 ± 4.29	0.79 ÷ 10.43
P-PO_4_	0.16 ± 0.11	0.06 ÷ 0.3	0.09 ± 0.02	0.07 ÷ 0.12	0.14 ± 0.13	0.06 ÷ 0.34

Periods: I, 15 January–15 April; II, 15 May–15 August; 15 September–15 December.

**Table 2:** Water parameters in the three periods at ST2. The results are shown as means ± standard deviation of three replicates. T (°C), Sal (‰), O_2_ (sat%), N-NH_4_, N-NO_2_, N-NO_3_, P-PO_4_ (μM/L).

	ST2					
	I		II		III	
Parameter	Mean ± SD	Range	Mean ± SD	Range	Mean ± SD	Range
T	16.39 ± 2.19	13.69 ÷ 19.07	23.38 ± 2.64	19.54 ÷ 25.23	19.45 ± 2.94	15.61 ÷ 22.47
SAL	31.62 ± 0.46	31.2 ÷ 32.2	31.3 ± 0.33	30.9 ÷ 31.7	31.02 ± 1.01	29.7 ÷ 32
pH	8.66 ± 0.13	8.51 ÷ 8.84	8.59 ± 0.12	8.42 ÷ 8.71	8.39 ± 0.16	8.2 ÷ 8.59
O_2_	106.6 ± 7.44	99.8 ÷ 114.8	82.02 ± 13.43	68.9 ÷ 94.1	92.62 ± 23.42	66.3 ÷ 120.3
N-NH_4_	2.33 ± 0.40	1.95 ÷ 2.73	1.64 ± 0.78	0.83 ÷ 2.64	2.91 ± 1.08	1.66 ÷ 4.05
N-NO_2_	0.36 ± 0.26	0.18 ÷ 0.76	0.32 ± 0.20	0.08 ÷ 0.52	0.71 ± 0.58	0.3 ÷ 1.56
N-NO_3_	7.55 ± 4.10	2.83 ÷ 11.22	1.73 ± 1.17	0.89 ÷ 3.4	3.33 ± 2.58	1.81 ÷ 7.2
P-PO_4_	0.17 ± 0.08	0.11 ÷ 0.3	0.20 ± 0.13	0.09 ÷ 0.41	0.39 ± 0.43	0.09 ÷ 1.03

Periods: I, 15 January–15 April; II, 15 May–15 August; 15 September–15 December.

**Table 3:** Water parameters in the three periods in ST3. The results are shown as means ± standard deviation of three replicates. T (°C), Sal (‰), O_2_ (sat%), N-NH_4_, N-NO_2_, N-NO_3_, P-PO_4_ (μM/L).

	ST3					
	I		II		III	
Parameter	Mean ± SD	Range	Mean ± SD	Range	Mean ± SD	Range
T	15.8 ± 2.55	13.24 ÷ 19.25	24.55 ± 3.13	20.23 ÷ 26.95	20.36 ± 3.54	16.16 ÷ 24.32
SAL	31.62 ± 0.46	31.2 ÷ 32.2	30.24 ± 1.08	28.68 ÷ 31.1	27.38 ± 2.07	24.73 ÷ 29.3
pH	8.66 ± 0.13	8.51 ÷ 8.84	8.43 ± 0.09	8.32 ÷ 8.55	8.47 ± 0.19	8.28 ÷ 8.71
O_2_	103.9 ± 2.11	101.5 ÷ 106.4	109.5 ± 25.57	78.2 ÷ 134.9	111.9 ± 26.30	75.5 ÷ 134.8
N-NH_4_	1.66 ± 0.41	1.11 ÷ 2.06	1.92 ± 0.55	1.33 ÷ 2.61	2.44 ± 1.03	1.55 ÷ 3.92
N-NO_2_	0.28 ± 0.15	0.12 ÷ 0.48	0.22 ± 0.10	0.1 ÷ 0.35	1.20 ± 1.14	0.19 ÷ 2.81
N-NO_3_	6.43 ± 4.00	1.64 ÷ 11.38	1.32 ± 0.32	0.88 ÷ 1.63	5.71 ± 4.91	0.99 ÷ 11.07
P-PO_4_	0.14 ± 0.02	0.11 ÷ 0.17	0.09 ± 0.04	0.05 ÷ 0.15	0.33 ± 0.56	0.02 ÷ 1.19

Periods: I, 15 January–15 April; II, 15 May–15 August; 15 September–15 December.

**Table 4:** Antibacterial activity of *G. gracilis* extracts against *B. subtilis* in agar disc diffusion assay. Inhibition halo is expressed in mm. The results are shown as means ± standard deviation of three replicates.

Organic Solvent	Amount of Extract (µg)	*B. subtilis* (mm)	Chloramphenicol (30 µg)
Ethanol	50	10 ± 0.00	13.56 ± 4.8
	100	14.6 ± 0.5	
	200	19 ± 1	
Methanol	50	7.6 ± 2	
	100	9.6 ± 0.5	
	200	12.6 ± 1.1	
Acetone	50	10 ± 0.00	
	100	11.6 ± 2.8	
	200	13.6 ± 3.5	
Chloroform	50	10.3 ± 0.5	
	100	15.6 ± 3	
	200	17.6 ± 2	
Diethyl ether	50	10 ± 1	
	100	10.6 ± 2	
	200	15.6 ± 3	

**Table 5:** Comparison among the extraction yield, total soluble carbohydrate (TSCC) and total phenolic content (TPC) revealed in different extracts of *G. gracilis* obtained by different organic solvents. TSCC and TPC measurements are reported as mean ± standard deviation (*n* = 3), on a dw basis.

Solvent	Extraction Yield *	TSCC **	TPC ***
Ethanol	9.80	553.24 ± 45.02 ^a^	2059.79 ± 94.41 ^a^
Methanol	1.37	159.14 ± 32.37 ^b^	2741.93 ± 219.52 ^b^
Acetone	0.51	42.83 ± 3.43 ^c^	810.32 ± 75.67 ^c^
Chloroform	0.61	0.35 ± 0.24 ^d^	35.90 ± 6.32 ^d^
Diethyl ether	0.39	0.36 ± 0.17 ^d^	28.83 ± 14.53 ^d^

* values of extraction yield are expressed as % of dry algal material; ** TSCC values are expressed as g GE/Kg of dry algal material; *** TPC values are expressed as mg GAE/100 g of dry algal material. ^a–d^ Different superscript letters in the same column indicate significantly different values (*p* < 0.05 by post hoc Tukey’s honestly significant difference (HSD) test); Same superscript letters in the same column indicate not significantly different values (*p* > 0.05 by post hoc Tukey’s HSD test).

**Table 6:** Levels (mg/100 g) of single polyphenols detected in ethanol, methanol and acetone extracts of *G. gracilis*. Data are reported as mean ± standard deviation (*n* = 3), on a dw basis.

Polyphenol	Ethanol Extract	Methanol Extract	Acetone Extract
Gallic acid	74.36 ± 4.14 ^a^	61.83 ± 4.08 ^b^	11.80 ± 1.75 ^c^
Protocatechuic acid	428.45 ± 26.60 ^a^	561.00 ± 33.76 ^b^	103.76 ± 10.55 ^c^
4-Hydroxybenzoic acid	124.93 ± 7.99 ^a^	167.00 ± 8.25 ^b^	59.36 ± 17.33 ^c^
Chlorogenic acid	35.60 ± 8.41 ^a^	69.56 ± 5.32 ^b^	46.49 ± 4.89 ^a^
Syringic acid	12.23 ± 2.61 ^a^	7.06 ± 1.27 ^b^	-
Caffeic acid	30.66 ± 2.30 ^a^	60.90 ± 7.00 ^b^	-
Coumaric acid	7.40 ± 0.87	-	-
Ferulic acid	5.60 ± 1.90 ^a^	12.80 ± 3.53 ^b^	-
Rutin	259.73 ± 13.45 ^a^	334.23 ± 25.66 ^b^	140.70 ± 19.08 ^c^
Hesperidin	539.33 ± 34.16 ^a^	741.30 ± 90.33 ^b^	203.43 ± 10.05 ^c^
Myricetin	174.23 ± 19.00 ^a^	285.96 ± 34.95 ^b^	81.60 ± 7.70 ^c^
Quercetin	157.43 ± 14.98 ^a^	206.63 ± 16.89 ^b^	73.60 ± 14.99 ^c^
Epicatechin	8.83 ± 1.90 ^a^	10.30 ± 1.15 ^a^	23.13 ± 1.58 ^b^
Epicatechingallate	13.13 ± 3.09 ^a^	10.83 ± 2.76 ^a^	35.16 ± 3.30 ^b^

^a–c^: Different superscript letters in the same column indicate significantly different values (*p* < 0.05 by post hoc Tukey’s HSD test); same superscript letters in the same column indicate not significantly different values (*p* > 0.05 by post hoc Tukey’s HSD test).

**Table 7:** Fatty acids (FA) composition determined in chloroform and diethyl ether extracts from *G. gracilis*. Data are reported on a dw basis, as mean gas chromatography–flame ionisation detection (GC-FID) peak area percent ± standard deviation of 3 replicate measurements.

FAME	Chloroform Extract	Diethyl Ether Extract
C12:0	0.81 ± 0.15	0.91 ± 0.32
C13:0	0.15 ± 0.05	0.09 ± 0.02
C14:0	4.76 ± 0.32	5.96 ± 1.25
C16:0	31.62 ± 1.44 *	41.71 ± 0.90 *
C18:0	3.92 ± 0.95	3.70 ± 0.39
C20:0	0.69 ± 0.04 *	0.43 ± 0.03 *
C22:0	0.40 ± 0.02	-
C24:0	0.12 ± 0.03 *	0.25 ± 0.03 *
*SFA*	42.36	52.81
C14:1n-9	0.15 ± 0.05	0.10 ± 0.02
C16:1n-7	2.18 ± 0.32	1.44 ± 0.33
C18:1n-9	10.33 ± 0.22 *	9.21 ± 0.38 *
*MUFA*	12.66	10.75
C18:2n-6	3.09 ± 0.21	2.05 ± 0.06
C18:3n-3	1.79 ± 0.20	0.70 ± 0.15
C20:2n-6	0.36 ± 0.16	-
C20:3n-6	1.34 ± 0.10	0.70 ± 0.07
C20:4n-6	38.33 ± 1.85 *	29.40 ± 1.35 *
C20:5n-3	1.62 ± 0.32	2.18 ± 0.12
C22:6n-3	-	0.80 ± 0.04
*PUFA*	46.55	35.85

* values in the same row marked by the asterisk are significantly different (*p* < 0.05) by the Student’s two-tailed *t*-test.
